# Global Epidemiology of Lung Cancer

**DOI:** 10.5334/aogh.2419

**Published:** 2019-01-22

**Authors:** Julie A. Barta, Charles A. Powell, Juan P. Wisnivesky

**Affiliations:** 1 Division of Pulmonary and Critical Care Medicine, Sidney Kimmel Medical College at Thomas Jefferson University, Philadelphia, PA, US; 2 Division of Pulmonary, Critical Care, and Sleep Medicine, Icahn School of Medicine at Mount Sinai, New York, NY, US; 3 Division of General Internal Medicine, Icahn School of Medicine at Mount Sinai, New York, NY, US

## Abstract

While lung cancer has been the leading cause of cancer-related deaths for many years in the United States, incidence and mortality statistics – among other measures – vary widely worldwide. The aim of this study was to review the evidence on lung cancer epidemiology, including data of international scope with comparisons of economically, socially, and biologically different patient groups. In industrialized nations, evolving social and cultural smoking patterns have led to rising or plateauing rates of lung cancer in women, lagging the long-declining smoking and cancer incidence rates in men. In contrast, emerging economies vary widely in smoking practices and cancer incidence but commonly also harbor risks from environmental exposures, particularly widespread air pollution. Recent research has also revealed clinical, radiologic, and pathologic correlates, leading to greater knowledge in molecular profiling and targeted therapeutics, as well as an emphasis on the rising incidence of adenocarcinoma histology. Furthermore, emergent evidence about the benefits of lung cancer screening has led to efforts to identify high-risk smokers and development of prediction tools. This review also includes a discussion on the epidemiologic characteristics of special groups including women and nonsmokers. Varying trends in smoking largely dictate international patterns in lung cancer incidence and mortality. With declining smoking rates in developed countries and knowledge gains made through molecular profiling of tumors, the emergence of new risk factors and disease features will lead to changes in the landscape of lung cancer epidemiology.

## Burden of Disease

Internationally, lung cancer continues to be the leading cause of cancer-related deaths in men and women [1]. A breakdown by level of economic development shows no differences in cancer deaths in men but a higher rate of lung cancer deaths in women in industrialized countries as compared with developing nations. Among females in developing countries, lung cancer deaths lag behind those due to breast cancer [2]. Lung cancer incidence and mortality are tightly linked to cigarette smoking patterns. As smoking rates peak – generally first in men, followed by women – lung cancer incidence and mortality rise in subsequent decades before declining following the initiation of comprehensive tobacco control programs [3,4,5]. These trends have occurred earlier in industrialized countries as compared with the developing world. In the United States (US) and the United Kingdom (UK), lung cancer incidence and mortality rates have in fact been falling since the 1990s. In contrast, emerging nations – including Brazil, Russia, India, China, and South Africa (BRICS) – continue to have high rates of cigarette smoking in both men and women. They exhibit a lower incidence of cancer but a higher mortality burden compared with developed countries. Reasons for these patterns include unequal access to healthcare leading to delayed diagnosis and treatment, environmental contamination, and sociocultural barriers [6].

### Industrialized Countries

In the US, the incidence of lung cancer in men peaked in the 1980s, followed by a subsequent decline, with similar patterns in women following 20 years later [7]. Thun et al. found that in the 1960s, the relative risk of lung cancer death in smokers versus non-smokers was more than four times higher in men than in women. In the 40 years since, women’s risk has risen markedly, becoming nearly identical to that of men [3,8]. Lung cancer deaths in men are now declining at an average of 2.9% annually with a percent decrease roughly double that of women [7]. With regard to differences between racial and ethnic groups, non-Hispanic whites and blacks have the highest incidence and death rates [9]. In particular, black men have the highest mortality, approximately double that of Asian Americans, the group with the lowest cancer-specific mortality [7,10]. These racial and ethnic disparities are largely due to differences in cigarette smoking prevalence, as well as lower rates of resection and higher probability of advanced stage at diagnosis in minorities [11,12,13].

**Figure 1:**
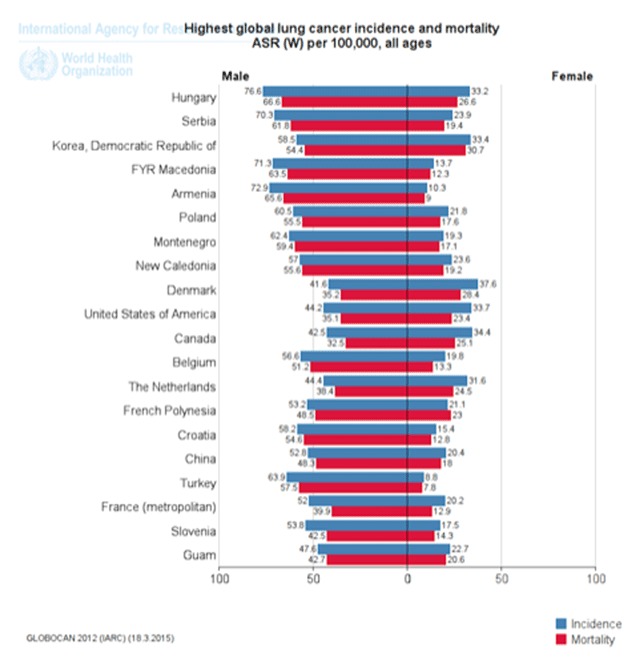
**Global lung cancer incidence and mortality.** Global age-standardized incidence and mortality rates for lung cancer, 20 countries with the highest rates internationally. Reproduced from GLOBOCAN 2012 data with permission.

The UK has similar smoking and lung cancer incidence trends to the US. Male smoking prevalence peaked in the 1940s to 1950s, followed by a peak in lung cancer incidence in the 1970s. Despite declining rates in both sexes, lung cancer remains the second most common malignancy in the UK [14]. Mainland Europe exhibits wide geographic variations in lung cancer incidence (Figure 1). In general, rates are highest in central and eastern Europe, but incidence throughout the continent has been declining in men since the early 1990s. Exceptions include Norway, Finland, Spain, and France, where lung cancer rates have remained stable. In women, rising lung cancer incidence has slowed in the US and UK, but rates continue to increase in central and eastern Europe [15,16,17,18,19,20]. These regional differences reflect earlier stages of the tobacco epidemic in countries such as Belarus, Hungary, Poland, and the Russian Federation [4,5,15,17]. Additionally, socioeconomic and educational inequalities, as well as diagnosis at later stages of disease, contribute to variability in lung cancer incidence and mortality within Europe [21,22]. Finally, similarly to the US, lung cancer survival is lower than that of any other common malignancies in Europe. EUROCARE-5 reported a mean five-year survival of 13% for all lung cancer patients diagnosed in 2000–2007, with a range from 9% in the UK and Ireland to 15% in central Europe [23,24].

In Asia, Japan has high incidence and mortality rates from lung cancer, comparable to those of the US and Europe [25]. Men have had a higher incidence of lung cancer than women since the 1970s and continue to comprise the majority of new lung cancer cases in Japan today, largely due to gender differences in smoking prevalence [26]. Conversely, mortality rates in women are lower in Japan than in other industrialized nations, perhaps due to the higher incidence of adenocarcinomas with mutations responsive to targeted therapies [27].

### Emerging Economies

Brazil, Russia, India, China, and South Africa are recognized by their large and fast-growing economies [28]. One of the few South American countries with a cancer registry is Brazil, where tobacco smoking peaked in the 1970s and lung cancer mortality in men peaked in 1993 and continues to rise among women [29,30]. Likewise, in the Russian Federation, all-cause mortality in men is largely attributed to very high rates (60%) of both smoking and alcohol consumption, which are much lower in women [6]. Accordingly, Russia has among the highest lung cancer mortality rate in men of all European countries but among the lowest in women. Mortality is now declining, after peaking in the early 1990s, but tobacco use remains a major barrier to effective cancer control [16]. Additional risk factors in Russia include environmental pollution and workplace exposures in nuclear facilities and asbestos mines [6].

Comparatively, lung cancer incidence and mortality rates in India are among the lowest in the world [4]. The most common cancers in men are head and neck, gastric, and esophageal cancers, attributed to high usage of smokeless tobacco; the most common cancers among women are cervical and breast. One study in northern India noted that squamous cell lung cancer was the most common histology overall and among smokers [31]. While cigarette smoking has a reported prevalence ranging from 28 to 57% among men, bidi smoking (hand-rolled tobacco) is the most commonly used (92%) tobacco product [32].

In 2005, the total number of new lung cancer cases in China was over 500,000. According to GLOBOCAN 2012, lung cancer is the most common malignancy and cause of cancer mortality in China, representing 21% of all cancers and 27% of all cancer-related deaths [6]. Lung cancer incidence and mortality is higher in eastern China and in urban areas, which has been attributed to westernization of lifestyle [33]. However, mortality rates are increasing faster in rural areas due to poor access to care [6]. Additionally, age-adjusted mortality rates are higher in Chinese men – 68% of whom are smokers – but lung cancer incidence rates are rising faster in women [34]. Risk factors among Chinese women include secondhand smoking, air pollution, and domestic use of biomass fuels [35].

### Developing Countries

Reporting of cancer epidemiology in Africa is limited by the lack of reliable registries. Among countries on the African continent as whole, both incidence and mortality rates are low – lung cancer was the fifth most common site of cancer in African men and not even in the top 10 for women. This is likely due to the low prevalence of smoking (10% in men and <2% in women) as well as the lower life expectancy of the population. Lung cancer does have a high incidence in certain regions including the northern African countries of Western Sahara, Morocco, Algeria, Tunisia, and Libya, and it is the leading cause of cancer death in men in northern and southern Africa [36].

South America has a wide range of lung cancer incidence across countries and markedly higher rates in men compared with women. The highest incidence and mortality in men can be found in Uruguay and in women of Venezuela and Argentina. Less populated countries such as Ecuador, Bolivia, and Guyana have very low age-standardized rates, just higher than those of central Africa and the Middle East [25].

The remainder of Asia has extremely diverse lung cancer incidences, which are nevertheless consistent within different regions. Asian countries closest to Eastern Europe such as Armenia, Turkey, and Kazakhstan have among the highest rates of lung cancer in the world. Korea and southeast Asia have slightly lower rates, and Middle Eastern countries including Yemen and Saudi Arabia have among the lowest lung cancer incidence rates in the world [25]. These notable regional differences reflect geographic trends in the tobacco epidemic [37].

## Histopathology

Lung cancer was traditionally classified into two primary groups, small versus non–small cell type. This grouping was progressively specified with the use of histopathologic features and immunohistochemical markers, and now inroads are being made in distinguishing invasive adenocarcinomas from pre-invasive lesions. Moreover, further knowledge about the molecular characteristics of lung cancers and the availability of targeted therapies has substantially impacted the classification of lung cancers.

### Histology

Adenocarcinoma is the most common histologic subtype of lung cancer in men and women [38]. Prior to the 1990s, squamous cell lung carcinoma was the most common histologic subtype, particularly among men. Since then, the incidence of adenocarcinoma rose to be greater than that of squamous cell carcinomas in the US, Canada, many European countries, and Japan [26,39]. However, this switch has not yet been observed in other countries such as Spain and the Netherlands [39]. The higher rates of adenocarcinoma relative to squamous and small cell lung cancer are greater in women [4]. Consequently, the proportion of adenocarcinomas is rising in many countries in parallel to increased incidence of lung cancer in women. These findings may reflect differences in the types of cigarettes (including filtered and low-tar versions) more frequently used by women as well as genetic predisposition and environmental exposures in female never-smokers [39].

In 2011, the International Association for the Study of Lung Cancer, American Thoracic Society, and European Respiratory Society proposed a new adenocarcinoma categorization based upon histological evidence of invasion. Preinvasive lesions are classified on a continuum from atypical adenomatous hyperplasia (AAH) to adenocarcinoma in situ (AIS), and minimally invasive adenocarcinoma (MIA) includes small (<3 cm) lesions with ≤5 mm of invasion. Invasive adenocarcinomas include a variety of patterns (e.g., lepidic predominant adenocarcinoma [LPA], acinar, papillary, micropapillary, and solid) characterized by tumor disruption of >5 mm of the alveolar basement membrane. This grouping correlates with clinical outcomes, with pre-invasive lesions having an indolent clinical course with almost 100% curability, in contrast to invasive carcinomas, which have a considerably worse prognosis [40]. Recent studies have shown AIS, MIA, and LPA to have a higher incidence in Japan compared with western populations [27].

Squamous cell lung cancer is the second most common subtype, comprising approximately 20% of primary lung neoplasms in the US. These tumors are distinguished histologically by squamous pearl formation, keratin production, and intercellular bridging. Historically, squamous cell lung cancer occurred more commonly as central lesions, but peripheral tumors are rising in incidence [41]. Small cell lung cancer, which has an aggressive clinical course, comprises 14% of lung cancers and typically presents as a perihilar mass with early and extensive lymph node metastases. It has a strong association with smoking history and commonly causes paraneoplastic syndromes. Less frequent histologic subtypes of lung cancer include large cell (3%), adenosquamous (1–2%), and carcinoid tumors (1–2%) [41].

### Molecular Markers

**Figure 2:**
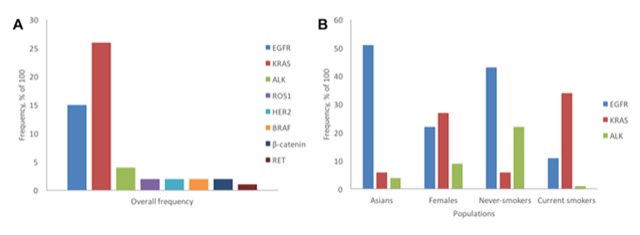
**Frequencies of common driver mutations in lung adenocarcinoma in the US and Europe.** By overall frequency (A) and population group (B). Data are derived from large clinicopathologic cohort studies published since 2008 and are representative of US and European populations.

The most common genetic alterations in lung adenocarcinoma are epidermal growth factor receptor (EGFR) and KRAS activating mutations (Figure 2). EGFR insertions and deletions are found in roughly 15% of lung adenocarcinomas in the US, with increased frequency in nonsmokers (43% vs. 11% in smokers) [42] and Asians (up to 60% in Asian women) [43]. In advanced stages of disease, this mutation predicts a more favorable prognosis and sensitivity to EGFR tyrosine kinase inhibitors (TKIs) such as erlotinib, gefitinib, and afatinib [44]. Conversely, KRAS mutations occur more commonly in smokers and appear to confer worse prognosis [42]. While no targeted therapeutics are currently available for this mutation, clinical trials are in progress to test drugs that target downstream effectors of activated KRAS [44]. Additional driver mutations in lung adenocarcinoma occur with a frequency of <1–4%, including ALK gene rearrangements, ROS1 translocations, HER2 mutations, BRAF mutations, and RET translocations. ALK rearrangement are clinically important however, as this mutation creates a fusion product, most frequently with EML4, which predicts sensitivity to ALK tyrosine kinase inhibitors such as crizotinib and ceritinib [44]. Additionally, ALK-positive tumors have been associated with acinar or solid histological patterns with signet ring features [45,46].

## Risk Factors and Environmental Exposures

The myriad risk factors for lung cancer most commonly include lifestyle, environmental, and occupational exposures. The roles these factors play vary depending on geographic location, sex and race characteristics, genetic predisposition, as well as their synergistic interactions.

### Cigarette Smoking, Secondhand Smoking, and E-cigarettes

Cigarette smoking is the most recognized risk factor for developing lung cancer. Since the Surgeon General’s report on smoking and health in 1964, at which time 52% of American men and 35% of American women were active smokers, the prevalence of cigarette smoking in US and consequently lung cancer has markedly decreased [47]. While nicotine itself is not carcinogenic, there be as many as 55 substances in cigarette smoke that have been deemed carcinogenic by the International Agency for Research on Cancer including polycyclic aromatic hydrocarbons and 4-(methylnitrosamino)-1-(3-pyridyl)-1-butanone (NNK). Their activation leads to the formation of DNA adducts and subsequent gene methylation, DNA sequence changes, DNA segment amplification or deletion, or whole chromosome gains or losses [48]. Relative risk of lung cancer in smokers as compared with smokers varies from 10- to 30-fold, and the degree of risk is dependent on number of cigarettes smoked daily and pack-years of smoking. Cigar and pipe tobacco smoking are also associated with increased odds of developing lung cancer [49].

Secondhand smoke exposure also leads to a dose-dependent risk of lung cancer. Öberg and colleagues studied the effects of environmental tobacco exposure (ETS) in 192 countries on six continents and found that 40% of children and 33%–35% of non-smokers are exposed to secondhand smoke. The highest rates were in Europe, the western Pacific, and parts of Southeast Asia; the lowest rates were found in Africa. Over 600,000 deaths worldwide, most of them in women, were attributable to secondhand smoking in 2004 [50]. Similar to Öberg’s finding of the relative risk for lung cancer in adult non-smokers exposed to secondhand smoke of 1.21 (95% confidence interval [CI]: 1.13–1.3), many regulatory bodies have reported an increase in lung cancer risk by 20 to 30% upon exposure to ETS [51,52,53,54,55]. The largest numbers of estimated deaths in adults attributable to secondhand smoke, however, are not due to lung cancer but rather ischemic heart disease and asthma [50].

Electronic cigarettes have sparked much recent controversy over potential risks from long-term use, as well as their role in smoking initiation and potentially cessation [56]. The 2012 National Youth Tobacco Survey found the prevalence of ever-use of e-cigarettes among middle and high school students in the US to be 6.8%, while prevalence among adults in the simultaneous National Adult Tobacco Survey was 1.9% [57,58]. Although conventional cigarette use is far higher (18% among adults), the incidence of e-cigarette use is rising rapidly and has been associated with higher odds of cigarette smoking and lower odds of abstinence [59,60]. Even more concerning, early research has shown that an e-cigarette vapor-conditioned media induced gene expression patterns in human bronchial epithelial cells concordant with that of cells exposed to a cigarette smoke-conditioned media [61].

### Domestic Biomass Fuels

Unprocessed biomass fuels, including wood, crop residues, dung, and coal, are used by approximately half of the world’s population for in-home cooking or heating, primarily in eastern and southern Asia [62]. Indoor emissions in these households contain high concentrations of polycyclic aromatic hydrocarbons, benzene, and other carcinogenic compounds [63]. Several studies have confirmed an increased lung cancer risk associated with biomass fuels, with one pooled analysis showing an odds ratio (OR) of 4.93 (95% CI: 3.73–6.52) among coal users in Asia when compared with nonsolid-fuel users [64,65]. A meta-analysis including subjects from Europe and North America, in addition to Asia, reported similar trends in lung cancer risk with exposure to coal, biomass, and mixed fuels [62]. Additional studies have reported increased risk with bituminous “smoky coal” use compared with anthracite-based “smokeless coal,” as well as higher risk with domestic exposures in smokers compared with nonsmokers [63,66].

### COPD and other Pulmonary Conditions

While epidemiological studies report that approximately 20–30% of smokers develop COPD and 10–15% develop lung cancer, COPD is by far the most common comorbidity in patients with lung cancer, with a varying prevalence between 30 and 70% [67]. A cohort of newly diagnosed lung cancer cases was reported to have a prevalence of COPD as high as six-fold greater than matched smokers without cancer [67]. Additional studies have shown factors such as increasing degree of airway obstruction, increasing age, lower body mass index, and a diffusing lung capacity of carbon monoxide < 80% to be associated with a diagnosis of lung cancer [68]. Furthermore, extent of emphysema on CT is an independent risk factor for lung cancer, as well as a predictor of cancer-specific mortality [69,70]. A recent pooled analysis of almost 25,000 cases from the International Lung Cancer Consortium also showed both lung cancer incidence and mortality to be significantly associated with emphysema [71,72]. Proposed mechanisms for the link between COPD and lung cancer include matrix remodeling and lung repair processes which lead to development of epithelial-mesenchymal transition and carcinogenesis. Additionally, several genome-wide association and candidate gene studies have identified associations between emphysema and lung cancer at several chromosomal loci, supporting that susceptibility to lung cancer may include COPD-related gene variants [73]. In a large meta-analysis, never-smokers with a history of chronic bronchitis, tuberculosis, or pneumonia were found to have an increased risk of lung cancer [71,74].

### Occupational Exposures

Exposure to asbestos is one of the most well-recognized occupational causes of lung cancer. Workers in asbestos mining and milling, shipbuilding, construction, textiles and insulation, and automobile repair are at the highest risk. Multiple mechanisms exist for carcinogenesis, including induction of oxidative damage and subsequent DNA deletions, somatic gene alterations, and enhanced delivery of tobacco carcinogens to the airway epithelium [75]. Markowitz and colleagues evaluated 2,377 North American insulators and found increased lung cancer risk to be associated with asbestos exposure (rate ratio: 3.6, 95% CI: 1.7–7.6) and asbestosis (rate ratio: 7.4, 95% CI: 4.0–13.7), with synergistic effects in smokers [76]. Diesel exhaust exposure has also been studied in trucking industry workers and coal miners. The SYNERGY project, a pooled analysis of 11 case-control studies conducted in Europe and Canada, which included 13,304 cases, showed that cumulative diesel exposure was associated with an increased lung cancer risk (OR: 1.31, 95% CI: 1.19–1.43) after controlling for other occupational exposures [77]. Similar findings were obtained in studies conducted in the US trucking and non-metal mining industries [78,79,80]. Other occupations with an increased incidence of lung cancer include coal-mining [81], asphalt paving with coal tar exposure [82], chimney sweeping [83], and painting [84] although the risk appears to be lower than that of asbestos and diesel exhaust. Other organic and metal exposures that have been associated with lung cancer include beryllium, cadmium, chromium, silica, formaldehyde, benzo[a]pyrene, nickel, hard metal dust, and vinyl chloride, which often act synergistically with tobacco smoking [84,85,86].

### Ambient Air Pollution and Other Environmental Exposures

European and American studies have evaluated the association of ambient air pollution with lung cancer risk. The ESCAPE study, an analysis of multiple cohorts from nine European countries, found particulate matter (PM) concentration in ambient air to be significantly associated with lung cancer risk (hazard ratio [HR]: 1.22, 95% CI; 1.03–1.45), particularly adenocarcinoma [87]. Studies in Canada [88], the Netherlands [89,90], and the UK [91] also found PM with median aerodynamic diameter less than 2.5 μm (PM_2.5_), in addition to nitrogen oxides, nitrogen dioxide, and sulfur dioxide to be associated with greater risk of lung cancer. In the US, analysis of the Cancer Prevention Study (CPS) II cohort found an increase in lung cancer mortality with increasing concentrations of PM_2.5_ in both non-smokers and smokers [92,93].

Arsenic occurring naturally in drinking water and food has been implicated in lung cancer [94,95]. Heck and colleagues evaluated 223 lung cancer cases and found an OR of 2.75 (95% CI; 1.00–7.57) for small cell and squamous cancers in subjects with increased toenail arsenic concentration [96]. Residential radon exposure is another known risk factor for lung cancer. An analysis of the CPS II cohort demonstrated a significant linear relationship between radon concentration and lung cancer mortality [97]. A Spanish case-control study found similar results, and also noted a strong interaction with tobacco (OR: 2.21 95% CI: 1.33–3.69 in non-smokers vs. OR: 73 95% CI: 20–268 in heavy smokers) [98].

### Diet and Nutrition

Fruit and vegetable consumption have been associated with decreased lung cancer risk in current smokers; ingestion of cruciferous vegetables, in particular, has been inversely associated with lung cancer risk [99,100]. An analysis of 264 lung cancer tissue samples showed differentially expressed miRNAs among subjects with intake of quercetin-rich versus quercetin-poor fruit and vegetables [101]. Many studies have attempted to evaluate the effects of vitamin levels and intake on lung cancer risk. Dietary and supplemental calcium intake has been shown to be inversely associated with lung cancer risk in female nonsmokers (HR: 0.66, 95% CI: 0.48–0.91), current smokers, particularly for lung adenocarcinoma [102,103]. In addition, total iron intake was inversely associated with lung cancer risk in women, while total magnesium intake increased risk in men and current smokers; no significant association was found between copper, selenium, and zinc with lung cancer risk. Johansson and colleagues found elevated serum vitamin B_6_ and methionine levels associated with a lower risk for lung cancer in never, former, and current smokers in Europe [104]. Two studies showed a protective effect in never-smoking women with vitamin D intake ≥ 400 IU/day and with supplemental soy intake [105,106]. Conversely, the Alpha-Tocopherol, Beta-Carotene Cancer Prevention Study, revealed a greater incidence of lung cancer and overall mortality in male smokers supplemented with 20mg b-carotene daily [107]. and prompted the US Preventive Services Task Force (USPSTF) to uphold their recommendation against the use of vitamin E for the lung cancer prevention. Moreover, the USPSTF recently concluded that there is insufficient evidence to recommend any vitamins, minerals, and multivitamin supplementation for lung cancer prevention [108].

### Genetic Factors

Genetic factors leading to increased susceptibility to lung cancer have been poorly studied. First-degree relatives of patients with lung cancer are at increased risk, even after adjusting for smoking habits [109]. A meta-analysis of 28 case-control studies and 17 observational cohort studies of individuals with positive family histories found a RR of 1.84 (95% CI: 1.64–2.05) for developing lung cancer [110]. Additionally, genome-wide association studies have suggested susceptibility loci on various chromosomes, including 5p15.33 and 3q28, among others, but later analyses have not replicated these results [111,112]. Other studies have identified polymorphisms in various enzymes such as cytochrome p450 enzymes and DNA repair genes [113], as well as germline mutations in the EGFR [114].

## Measures of Lung Cancer Risk and Impact on Early Diagnosis

Except for smoking cessation, perhaps the highest reduction in lung cancer mortality rates is related to diagnosis at early stage followed by surgical resection. While chest X-rays and sputum cytology screening have not shown a benefit, early detection via low-dose chest tomography (CT) is now endorsed by the USPSTF [115]. The National Lung Screening Trial (NLST) compared annual screening by low-dose chest tomography (LDCT) with chest X-ray for three years at 33 US medical centers in 53,454 high-risk subjects 55 to 74 years of age with at least 30 pack-years of smoking and found a 20% lung cancer mortality reduction [116]. Ongoing European trials will provide additional critical information regarding the potential benefits of lung cancer screening [117,118]. However, CT screening is associated with high rates of positive findings and may lead to identification of some lung cancers with low aggressiveness [119]. Thus, better risk stratification using prediction models or biomarkers of lung cancer risk, as well as a better understanding of the biologic characteristics of aggressive cancers is required to maximize the benefit of screening.

Even within smokers, lung cancer risk varies considerably based on factors such as age, quantity and duration of smoking, and environmental exposures [3]. There is an ongoing effort to stratify lung cancer risk to identify individuals best suited for lung cancer screening or refine eligibility for prevention trials. Several models based on sociodemographic characteristics, smoking, and other risk factors have been empirically derived using relatively large cohorts [120,121]. Among these, the Bach model was developed using information from 18,172 subjects in the Carotene and Retinol Efficacy Trial, which followed heavy smokers and asbestos-exposed workers from 1989 to 1996 [122]. This model was validated in the Alpha-Tocopherol Beta-Carotene Cancer Prevention Study and was found to underestimate 10-year absolute lung cancer risk while having a discriminatory power comparable to breast cancer risk models [123]. Likewise, the Spitz model was derived and validated in a cohort of 1,851 lung cancer patients and 2,001 matched controls from a single tertiary center. Risk factors among current and former smokers, including exposure to environmental tobacco smoke, dust, fumes, chemicals, history of emphysema, and family history of cancer, predict an increased risk for cancer [124]. Finally, the Liverpool Lung Project model, validated in multiple independent populations, is based on data about smoking duration, history of pneumonia or cancer, family history of lung cancer, and asbestos exposure to predict five-year lung cancer risk [125,126].

Recent research has focused on identifying biomarkers of lung cancer risk, aggressive behavior among early cancers, and prognosis. These biomarkers may be produced by neoplastic cells themselves, the tumor microenvironment, or the host. A variety of methods to identify biosignatures utilizing tissue- and biofluids-based assays have been tested and include genome-wide association studies (GWAS), epigenetics, microRNA, and proteomics [127]. Using GWAS, several single nucleotide polymorphisms (SNPs) have been identified on specific chromosomal loci – such as the 15q25 locus – that are associated with tobacco exposure and lung cancer [128]. It remains to be seen whether these SNPs can be utilized in the clinical setting to assess lung cancer risk. Recent epigenetic studies have shown promise in risk stratification, with one case-control study using methylation of genes in sputum to identify asymptomatic patients with stage I lung cancer [129]. Several studies have assessed circulating microRNA biomarkers. A 34-microRNA signature was reported to identify early-stage NSCLC patients with 80% accuracy [130], while others have been shown to predict recurrent disease [131] in plasma, surgically resected specimens, and in small biopsies [132]. Similarly, a 24-micro RNA signature [133], validated in a correlative study within the MILD CT-screening trial, was found to reduce by five-fold the false positive rate after low-dose CT [134]. Finally, serum proteomic signatures have been integrated with CT imaging features to predict lung cancer diagnosis in subjects with indeterminate lung nodules [135]. A seven-autoantibody signature has been shown to have high specificity for lung cancer-associated antigens for early detection of lung cancer in a high-risk population, and also in distinguishing benign from malignant disease in CT-detected lung nodules; prospective validation is ongoing [136]. Ajona and colleagues demonstrated that C4d, a degradation product of complement activation, was elevated in tumors, bronchoalveolar lavage fluid, and plasma samples from stage I–II lung cancer patients compared to controls. Additionally, C4d levels were associated with worse survival and increased lung cancer risk in screen-detected lung nodules [137]. Despite a substantial progress in biomarker discovery, challenges that remain include selection of appropriate candidate signatures based on tumor-specificity and high-throughput approaches, genetic heterogeneity of tumors, and reproducibility in external validation studies [138,139].

## Special Populations

Lung cancer incidence is rising in women and has in fact more than doubled since the mid1970s. This increase has been attributed to increased susceptibility in women compared with men, although studies have found conflicting results. Data from the UK’s Health Improvement Network showed that female heavy smokers (>20 cigarettes daily) had a greater odds of developing lung cancer than men with comparable smoking histories, with an adjusted OR of 19.2 (95% CI: 17.1–21.3) in women versus 13.0 (95% CI: 11.7–14.5) in men [140]. However, a large prospective cohort study in the US disputed this increased susceptibility to lung cancer given equal smoking exposure [141,142]. Rising rates of lung cancer in women have also been attributed to genetic variants, environmental exposures, hormonal factors, and oncogenic viruses [141,143]. The role of reproductive and hormonal factors remains controversial, with mixed results in studies evaluating associations between parity, age at menarche, and menopause. Although multiple case-control studies report increased lung cancer risk with exogenous hormone therapy [144,145,146], prospective cohort studies show equal lung cancer rates after adjusting for smoking rates [144,145,147,148,149,150]. HPV infection has been implicated in the pathogenesis of lung cancer in women in Asia, but in the US, infection rates in tumors have been much lower [141], and one Finnish study of 311 women with lung cancer, found no evidence of increased risk for lung cancer with HPV 16 and 18 type-specific infections among both nonsmokers and smokers [151]. Finally, with regard to diet, a Japanese prospective cohort study of 126 newly diagnosed women with lung cancer found only a trend toward an association with plasma genistein, an isoflavone found in soy and previously shown to act as estrogen agonists and antagonists [152]. Although adenocarcinoma is the most common histologic subtype in both genders, women have an even higher predominance of this cell type and have a higher likelihood of developing adenocarcinoma in situ, a preinvasive lesion [141]. Additionally, several gender differences in lung cancer mutations have been described. EGFR mutations are more prevalent in women, especially in non-smokers [153]. In particular, the L858R mutation has been shown to be associated with genetic polymorphisms related to estrogen biosynthesis and metabolism in never-smoking females with lung adenocarcinomas [153]. In one large study of the molecular epidemiology of lung cancer, the KRAS G12C mutation, the most common G>T transversion mutation in smokers, was more frequent in women, particularly of younger age [42]. Additionally, women with lung cancer have been shown in multiple studies to have better survival rates than men across different age groups, disease stage, and treatment types [3,141,154].

Approximately 10 to 20% of lung cancers occur in never-smokers with a much higher incidence in women than in men [155]. In fact, in South Asia, it is estimated that 83% of women with lung cancer may be never-smokers [156]. It is unclear if never-smoking Asian women who emigrate to the US and adopt western lifestyles continue to have elevated risks of lung cancer, although prior literature in gastric cancer appears to suggest that risk is environmental in nature [157]. Additional risk factors thought to contribute to lung cancer in never-smokers are environmental and occupational exposures and genetic susceptibility [156]. Never-smoking women present at a more advanced stage, and studies from Asia have reported an earlier age at diagnosis compared with smokers. However, in the US and Europe, never-smokers and ever-smokers are diagnosed with lung cancer at a similar age [156]. With regard to histology, never-smokers have a higher prevalence of adenocarcinoma [156]. Additionally, while genomic mutations occur more frequently in current and former smokers compared with never-smokers, the latter have a higher prevalence of driver mutations including EGFR and ALK-EML4 [158,159]. The EGFR mutation via an exon 19 deletion or exon 21 mutation is found in up to 40–60% of never-smokers [42,43,160]. HER2 mutations, a member of the EGFR family, also occurs predominantly in never-smokers [44]. In contrast, KRAS and BRAF mutations occur mainly in former and current smokers [161]. Moreover, approximately two-thirds of patients with ALK-EML4 rearrangements are never-smokers [45,46,162]. Finally, never-smokers exhibit improved survival compared with smokers, even after adjusting for known prognostic factors [156].

Since the advent of antiretroviral therapy in the 1990s, cancer mortality among human immunodeficiency virus (HIV) patients has shifted from acquired immunodeficiency syndrome (AIDS)-related malignancies to solid-organ cancers. Lung cancer incidence is increased two- to four-fold in the HIV-infected population compared with the general population and occurs with a lower pack-year rate of smoking [163,164,165]. Using the Veterans Affairs Central Cancer Registry, Sigel et al. found HIV to be an independent risk factor for the development of lung cancer, with an incidence rate ratio of 1.7 (95% CI: 1.5–1.9) [166]. Multiple cohorts have shown that there is no difference in age, stage at presentation, or histology between HIV-positive and -negative patients [166,167]. Lung cancer has also been found to be more aggressive in HIV patients, and positive HIV status has been associated with greater risk of lung cancer-specific mortality [168]. However, cancer-related survival has been shown to be better in patients with CD4 counts > 200 cells/mL [168,169,170]. Additionally, in subjects undergoing surgical resection, HIV patients have been found to have worse post-operative pulmonary and infectious complications as well as shorter median time to cancer progression [169].

## Conclusions

This review has examined international trends in lung cancer epidemiology. Emerging economies and developing countries face many challenges in initiating tobacco cessation campaigns while also addressing environmental risk factors and cultural barriers. Over the past three to four decades, in contrast, industrialized nations have seen large declines in cigarette smoking and, consequently, lung cancer. However, despite knowledge gains in tumor biology that have led to targeted therapies, mortality from lung cancer remains high for most patients around the world. Future directions must include improvements in early detection and technological advances in genomics and genetics to achieve a more personalized approach to therapy and ultimately improve lung cancer survival.
